# Supplementary material for: MetaRibo-Seq measures translation in microbiomes
Source: Nat Commun. 2020 Jun 29;11:3268. doi: 10.1038/s41467-020-17081-z (PMC7324362; doi:10.1038/s41467-020-17081-z)
Supplement: Supplementary file 10 — Supplementary Data 7 [file 41467_2020_17081_MOESM10_ESM.zip › File2/Confidence_High_Taxonomy/319484_out.krona.html]

Javascript must be enabled to view this page.

members
magnitude
magnitudeUnassigned
count
unassigned
taxon
rank

319484\_out

13

superkingdom
2
13

1239
phylum
13

13
class
186801

186802
order
13

13
186803
family

13
841
genus


SRS011084\_contig\_number\_19449SRS011405\_contig\_number\_15649SRS012849\_contig\_number\_23361SRS015264\_contig\_number\_9990SRS019161\_contig\_number\_37764SRS048870\_contig\_number\_3427SRS063040\_contig\_number\_44235SRS065504\_contig\_number\_29907SRS077194\_contig\_number\_9949SRS104693\_contig\_number\_7535SRS143148\_contig\_number\_16601SRS144506\_contig\_number\_47152SRS148159\_contig\_number\_35984
13
species
360807
